# Variant analysis of 92 Chinese Han families with hearing loss

**DOI:** 10.1186/s12920-022-01158-3

**Published:** 2022-01-21

**Authors:** Xiaohua Jin, Shasha Huang, Lisha An, Chuan Zhang, Pu Dai, Huafang Gao, Xu Ma

**Affiliations:** 1 Graduate School of Peking Union Medical College, Beijing, 100730 China; 2 National Research Institute for Family Planning, National Human Genetic Resources Center, Beijing, 100081 China; 3 Department of Otolaryngology Head and Neck Surgery, Chinese People’s Liberation Army (PLA) General Hospital, Beijing, 100853 China; 4 Center for Medical Genetics, Gansu Provincial Clinical Research Center for Birth Defects and Rare Diseases, Gansu Provincial Maternity and Child-Care Hospital, Lanzhou, 730050 Gansu China

**Keywords:** Hearing loss, Genetic heterogeneity, Molecular diagnosis, Genetic counseling

## Abstract

**Background:**

Hearing loss (HL) is the most frequent sensory deficit in humans, HL has strong genetic heterogeneity. The genetic diagnosis of HL is very important to aid treatment decisions and to provide prognostic information and genetic counseling for the patient’s family.

**Methods:**

We undertook pedigree analysis in 92 Chinese non-syndromic HL patients by targeted next-generation sequencing and Sanger sequencing.

**Results:**

Among the 92 HL patients, 18 were assigned a molecular diagnosis with 33 different variants in 14 deafness genes. Eighteen of the variants in 12 deafness genes were novel. Variants in *TMC1*, *CDH23*, *LOXHD1* and *USH2A* were each detected in two probands, and variants in *POU3F4*, *OTOA*, *GPR98*, *GJB6*, *TRIOBP*, *SLC26A4*, *MYO15A*, *TNC*, *STRC* and *TMPRSS3* were each detected in one proband.

**Conclusion:**

Our findings expand the spectrum of deafness gene variation, which will inform genetic diagnosis of deafness and add to the theoretical basis for the prevention of deafness.

## Background

Hearing loss (HL) is the most frequent sensory deficit in humans, with a prevalence of approximately 1/1000 in newborns [1, 2]. Hearing loss in approximately 50% to 60% of individuals is caused by genetic factors [3]. Among these, approximately 70% are non-syndromic HL (NSHL), in which the hearing impairment is the only distinctive clinical feature, while 30% of HL patients are syndromic with other abnormalities [4]. NSHL also has strong genetic heterogeneity.

The genetic diagnosis of NSHL is very important to aid treatment decisions and to provide prognostic information and genetic counseling for the patient’s family [5, 6]. The genetic mode of NSHL inheritance can be autosomal recessive, autosomal dominant, mitochondrial, or X/Y-linked. The development of molecular diagnostic technology has greatly reduced the cost of testing, and next-generation sequencing (NGS) has become an effective way of providing comprehensive and efficient diagnosis for NSHL [7]. To date, 224 genes have been reported to be associated with hearing loss (https://morl.lab.uiowa.edu/genes-included-otoscope-v9). Sixty-six are autosomal dominant, 117 are autosomal recessive, 21 are autosomal dominant/autosomal recessive, 9 are mitochondrial, and 5 are X-linked. However, most of the variations in these genes are rare and have only been reported in one or a few families [8].

Molecular epidemiological studies have found that the three common deafness genes *GJB2*, *SLC26A4*, and mtDNA *12S rRNA* accounted for 30–50% of congenital HL [9]. In China, nine variants in four genes are the most common causes of NSHL, including c.235delC (18.3%), c.299_300delAT (5.6%), c.176del16 (1.8%) and c.35delG (0.14%) of *GJB2*; c.919-2A>G (15.4%) and c.2168A>G (1.08%) of *SLC26A4*; m.1555A>G (1.76%) and m.1494C>T (0.16%) of mtDNA *12S rRNA*; c.538C>T (0.41%) of *GJB3* [10–12]. A large neonatal cohort study in Beijing, China, showed that the heterozygous carrier rate of *GJB2* gene was 2.3%, the *SLC26A4* was 1.6%, the mtDNA *12S rRNA* was 0.2% and the *GJB3* was 0.3% [12].

Here, we recruited 92 Chinese Han NSHL families, who were confirmed not to carry the common HL variants in *GJB2*, *SLC26A4* and *MT-RNR1*. Targeted NGS for known deafness genes was performed on the probands of each family to search for the genetic etiology of HL.

## Methods

### Recruitment of patients

92 patients with non-syndromic deafness were clinically diagnosed with bilateral sensorineural hearing loss at the Chinese People's Liberation Army (PLA) General Hospital (Beijing). Audiological tests were performed in the hearing center of the Chinese PLA General Hospital. Tests included pure-tone audiometry (or behavioral audiometry) for patients > 4 years old and multiple-frequency auditory steady-state evoked response (ASSR) tests for patients ≤ 4 years old [13]. All the probands were from non-consanguineous families. They were aged from 6 months to 54 years, and the age of onset ranged from birth to 22 years (Table 2).

### Genomic DNA preparation

Blood samples (1–2 mL) were collected from the probands and their parents. Genomic DNA was extracted using a Tiangen DNA extraction kit (Tiangen Biotech, Beijing, China) according to the manufacturer’s instructions and quantified spectrophotometrically by NanoDrop 2000 manufacturer (ThermoScientific, USA).

### Targeted -NGS and Sanger sequencing

Targeted capture of candidate disease genes (Table 1) was performed using the GenCap™ Custom Enrichment kit (MyGenostics, Beijing, China). Data analysis and bioinformatics analysis were performed according to method described by previous study [6]. Candidate variants were confirmed in the proband’s parents in each family by Sanger sequencing. The PCR products were bi-directionally sequenced using the BigDye Terminator v3.1 Cycle Sequencing Kit (Applied Biosystems, USA) on an ABI 3500DX Genetic Analyzer (Applied Biosystems, USA) after purification of the products in 2% agarose gels by using a Tiangen Midi Purification kit (Tiangen Biotech, Beijing, China).

**Table 1:** Genes in the hearing loss target-NGS panel

*ACTG1*	*CLPP*	*FOXI1*	*KRT9*	*PCDH9*	*SOX3*
*ALX3*	*CLRN1*	*FREM1*	*LAMA3*	*PDZD7*	*STRC*
*ATP6V0A1*	*COCH*	*FXN*	*LARS2*	*PJVK*	*STRN3*
*ATP6V0A2*	*COL11A1*	*GATA3*	*LHFPL5*	*PMP22*	*TARID*
*ATP6V0A4*	*COL11A2*	*GIPC3*	*LOXHD1*	*PNPT1*	*TBC1D24*
*ATP6V0B*	*COL1A1*	*GJB1*	*LRTOMT*	*POLR1C*	*TCIRG1*
*ATP6V0C*	*COL1A2*	*GJB2*	*MARVELD2*	*POLR1D*	*TCOF1*
*ATP6V0D1*	*COL2A1*	*GJB3*	*MIR96*	*POU3F4*	*TECTA*
*ATP6V0D2*	*COL4A3*	*GJB6*	*MITF*	*POU4F3*	*TERF2IP*
*ATP6V0E1*	*COL4A4*	*GLYAT*	*MPZ*	*PROK2*	*TIMM8A*
*ATP6V0E2*	*COL4A5*	*GPR98*	*MSRB3*	*PROKR2*	*TJP2*
*ATP6V1A*	*COL4A6*	*GPSM2*	*MYH14*	*PRPS1*	*TMC1*
*ATP6V1C1*	*COL9A1*	*GRHL2*	*MYH9*	*PTPN11*	*TMEM126A*
*ATP6V1C2*	*COL9A2*	*GRIA3*	*MYO15A*	*PTPRQ*	*TMIE*
*ATP6V1D*	*CRYM*	*GRXCR1*	*MYO1A*	*PTPRR*	*TMPRSS3*
*ATP6V1E1*	*DIABLO*	*HARS*	*MYO1C*	*PXMP2*	*TMPRSS4*
*ATP6V1E2*	*DIAPH1*	*HARS2*	*MYO1E*	*RDX*	*TNC*
*ATP6V1F*	*DIAPH3*	*HGF*	*MYO3A*	*RPGR*	*TPRN*
*ATP6V1G1*	*DSPP*	*HMX1*	*MYO6*	*SALL1*	*TRIOBP*
*ATP6V1G2*	*DTD1*	*HOXA2*	*MYO7A*	*SALL4*	*TRMU*
*ATP6V1G3*	*ECM1*	*HSD17B4*	*NDP*	*SEC23A*	*TSPEAR*
*ATP6V1H*	*EDN3*	*HSPA1A*	*NDRG1*	*SEMA3E*	*TYR*
*BCL2L2*	*EDNRB*	*HSPA1B*	*NEFL*	*SERPINB6*	*USH1C*
*BSND*	*ELMOD3*	*HSPA1L*	*NELL2*	*SIAH2*	*USH1G*
*C19orf83*	*EML2*	*IFNLR1*	*NF2*	*SIX1*	*USH2A*
*CABP2*	*ESPN*	*IL13*	*OPA1*	*SIX5*	*WFS1*
*CAT*	*ESRRB*	*ILDR1*	*OTOA*	*SLC17A8*	*WHRN*
*CCDC50*	*EYA1*	*KARS*	*OTOF*	*SLC19A2*	*YARS2*
*CDH23*	*EYA4*	*KCNE1*	*OTOG*	*SLC26A4*	
*CEACAM16*	*FGF3*	*KCNJ10*	*OTOGL*	*SLC26A5*	
*CHD7*	*FGF8*	*KCNQ1*	*P2RX2*	*SMAD4*	
*CIB2*	*FGFR1*	*GSDME*	*PABPN1*	*SMPX*	
*CKMT1A*	*FGFR3*	*KCNQ4*	*PAX3*	*SNAI2*	
*CLDN14*	*FLNA*	*KITLG*	*PCDH15*	*SOX10*	

### Bioinformatics analysis

Variants are described according to the nomenclature recommended by the Human Genome Variation Society (www.hgvs.org/). Variants were annotated using ANNOVAR (https://annovar.openbioinformatics.org/en/) and filtered according to their predicted effects and allele frequencies in the public database, gnomAD (http://gnomad.broadinstitute.org/). Novel variants were checked in the Human Gene Variant Database (HGMD; www.hgmd.cf.ac.uk/), ClinVar database (www.ncbi. nlm.nih.gov/clinvar/) and gnomAD database. We use PolyPhen2(Polymorphism Phenotyping, http://genetics.bwh.harvard.edu/pph2) and PROVEAN (http://provean.jcvi.org/index.php) tools to assess the possible functional role of the novel variant. The conservativeness of the novel site is evaluated on the UCSC website (https://genome.ucsc.edu/). InterVar (http://wintervar.wglab.org/) was used to evaluate the pathogenicity of all variants according to the standards and guidelines of the American College of Medical Genetics and Genomics (ACMG) [14].

## Results

### Variant analysis

Among 92 probands analyzed, we determined a genetic diagnosis in 18, and all the 92 probands were from non-consanguineous families. Three modes of inheritance were observed, including 15 autosomal recessive cases, 2 autosomal dominant cases, and 1 X-linked recessive case (Table 2). Fourteen deafness gene variants were detected. Those in *TMC1*, *CDH23*, *LOXHD1* and *USH2A* were each detected in two probands, and those in 10 other deafness genes were each detected in one proband (Table 2). The 18 probands carried 33 different variants (Table 2), of which 18 were novel, accounting for 54.5% of the total variants (18/33). These 33 variants included six different variant types, including 11 missense variants (33.3%, 11/33), 9 nonsense variants (27.3%, 9/33), 8 frameshift variants (24.2%, 8/33), 1 non-frameshift variant (3.0%, 1/33), 3 splice site variants (9.1%, 3/33), and 1 copy number variation (CNV) variant (3.0%, 1/33).

**Table 2:** Variants analysis of the 18 HL patients in this study

Patient ID	Sex	Age	Age of diagnoses	Gene	Inheritance	NM Transcrip	Nucleotide change	Amino acid change	Variant type	gnomAD allele frequency	Reported or not	Disease	Pedigree
12471	M	5.5Y	2.5y	*POU3F4*	XLD	NM_000307	c.881A>G	p.E294G	Missense	–	Novel	X-linked deafness-2	Nuclear family
12480	F	4Y	Birth	*USH2A*	AR	NM_206933	c.8167C>T	p.R2723X	Nonsense	–	[15]	Usher syndrome 2A	Sporadic case
				*USH2A*	AR	NM_206933	c.99_100insT	p.R34fs	Frameshift	0.00003	[16]		
12513	F	6Y	5Y	*OTOA*	AR	NM_144672	c.774A>C	p.L258F	Missense	–	Novel	Autosomal recessive deafness-22	Nuclear family
				*OTOA*	AR	NM_144672	c.1764delC	p.F588fs	Frameshift	0.000012	[17]		
12601	F	33Y	33Y	*GPR98*	AR	NM_032119	c.12640C>T	p.Q4214X	Nonsense	–	Novel	Usher syndrome 2C	Sporadic case
				*GPR98*	AR	NM_032119	c.14404C>T	p.R4802X	Nonsense	–	[18]		
12606	M	8Y	8Y	GJB6	AD	NM_001110219	c.228delC	p.W77Gfs	Frameshift	0.00000725	Novel	Autosomal dominant deafness-3B	Nuclear family
12622	F	5Y	1Y	*CDH23*	AR	NM_022124	c.805C>T	p.R269W	Missense	0.000007	Novel	Autosomal recessive deafness-12	Nuclear family
				*CDH23*	AR	NM_022124	c.5994delG	p.V1998fs	Frameshift	–	Novel		
12712	F	28Y	25Y	*TRIOBP*	AR	NM_001039141	c.1960C>T	p.R654X	Nonsense	0.000007	Novel	Autosomal recessive deafness-28	Sporadic case
				*TRIOBP*	AR	NM_001039141	c.5968delT	p.F1990fs	Frameshift	–	Novel		
12751	F	10Y	1Y	*SLC26A4*	AR	NM_000441	c.589G>A	p.G197R	Missense	0.000004	[19, 19]	Pendred syndrome	Nuclear family
				*SLC26A4*	AR	NM_000441	c.1238A>G	p.Q413R	Missense	0.000007	[20, 20]		
12761	M	3Y	6M	*CDH23*	AR	NM_022124	c.5957T>C	p.L1986P	Missense	–	Novel	Autosomal recessive deafness-12	Nuclear family
				*CDH23*	AR	NM_022124	c.6830C>A	p.A2277D	Missense	–	Novel		
12792	F	24Y	6Y	*USH2A*	AR	NM_206933	c.8559-2A>G	Splice	Splice site	–	[22]	Usher syndrome 2A	Sporadic case
				*USH2A*	AR	NM_206933	c.3791delC	p.S1264fs	Frameshift	–	Novel		
12802	F	5Y	Birth	*MYO15A*	AR	NM_016239	c.6611G>A	p.R2204H	Missense	–	Novel	Autosomal recessive deafness-3	Sporadic case
				*MYO15A*	AR	NM_016239	c.10251_10253del	p.3417_3418del	nonframeshift	0.000007	[22, 22]		
12812	F	27Y	6Y	*LOXHD1*	AR	NM_144612	c.2295G>A	p.W765X	Nonsense	–	Novel	Autosomal recessive deafness-77	Sporadic case
				*LOXHD1*	AR	NM_144612	c.134A>C	p.Y45S	Missense	–	Novel		
12834	M	31Y	31Y	*TMC1*	AR	NM_138691	c.236+1G>C	Splice	Splice site	–	[23]	Autosomal recessive deafness-7	Sporadic case
				*TMC1*	AR	NM_138691	c.741+2T>C	Splice	Splice site	–	Novel		
12852	M	4Y	1Y	*LOXHD1*	AR	NM_144612	c.6355delG	p.A2119fs	Frameshift	–	Novel	Autosomal recessive deafness-77	Nuclear family
				*LOXHD1*	AR	NM_144612	c.5888delG	p.G1963fs	Frameshift	0.0000065	ClinVar		
12918	M	54Y	54Y	*TNC*	AD	NM_002160	c.1641C>A	p.C547X	Nonsense	0.000004	Novel	Autosomal dominant deafness-56	Sporadic case
12929	F	3Y	1Y	*TMC1*	AR	NM_138691	c.100C>T	p.R34X	Nonsense	0.000004	[24, 24]	Autosomal recessive deafness-7	Nuclear family
				*TMC1*	AR	NM_138691	c.1810C>T	p.R604X	Nonsense	0.00001	[26]		
12932	M	11Y	10Y	*STRC*	AR	NM_153700	c.4778C>T	p.A1593V	Missense	0.000004	Novel	Autosomal recessive deafness-16	Nuclear family
				*STRC*	AR	NM_153700	CNV	–	CNV	–	[27, 28]		
12933	M	3Y	3Y	*TMPRSS3*	AR	NM_024022	c.916G>A	p.A306T	Missense	0.000145	[29, 30]	Autosomal recessive deafness-8	Nuclear family
				*TMPRSS3*	AR	NM_024022	c.271C>T	p.R91X	Nonsense	0.000004	[27]		

M, male; F, female; Y, year’s old; M, month; –, not included in the gnomAD database

According to the ACMG guidelines and InterVar sofware, 10 of the novel variants were categorized as “pathogenic”, and 8 were “likely pathogenic” (Table 3).

**Table 3:** Pathogenicity analysis of novel variants

Gene	Nucleotide change	Amino acid change	PolyPhen2 Result (Score)	PROVEN Result (Score)	Pathogenicity	Conservative	ACMG evidence
*CDH23*	c.805C>T	p.R269W	PD(1.000)	N(− 2.154)	LP	Yes	PM1, PM2, PP1, PP3
*CDH23*	c.5994delG	p.V1998fs	–	–	P	Yes	PVS1, PM2, PM4
*CDH23*	c.5957T>C	p.L1986P	PD(1.000)	N(− 0.743)	LP	Yes	PM1, PM2, PP1, PP3
*CDH23*	c.6830C>A	p.A2277D	PD(0.999)	N(− 2.146)	LP	Yes	PM1, PM2, PP1, PP3
*GJB6*	c.228delC	p.W77Gfs	–	–	P	Yes	PVS1PM2 PM4
*GPR98*	c.12640C>T	p.Q4214X	–	–	P	Yes	PVS1, PM2, PP3
*LOXHD1*	c.2295G>A	p.W765X	–	–	P	Yes	PVS1, PM2, PP3
*LOXHD1*	c.134A>C	p.Y45S	PD(0.999)	D(− 5.352)	LP	Yes	PM1, PM2, PP1, PP3
*LOXHD1*	c.6355delG	p.A2119fs	–	–	P	Yes	PVS1, PM2, PM4
*MYO15A*	c.6611G>A	p.R2204H	PD(1.000)	D(− 4.955)	LP	Yes	PM1, PM2, PP1, PP3
*OTOA*	c.774A>C	p.L258F	PD(1.000)	N(− 2.150)	LP	Yes	PM2, PM3, PP1, PP3
*POU3F4*	c.881A>G	p.E294G	PD(1.000)	D(− 7.000)	LP	Yes	PM1, PM2, PP1, PP3
*STRC*	c.4778C>T	p.A1593V	PD(0.999)	D(− 3.044)	LP	Yes	PM2, PM3, PP1, PP3
*TMC1*	c.741+2T>C	splice	–	–	P	Yes	PVS1, PM2, PM4
*TNC*	c.1641C>A	p.C547X	–	–	P	Yes	PVS1, PM2, PP3
*TRIOBP*	c.1960C>T	p.R654X	–	–	P	Yes	PVS1, PM2, PP3
*TRIOBP*	c.5968delT	p.F1990fs	–	–	P	Yes	PVS1, PM2, PM4
*USH2A*	c.3791delC	p.S1264fs	–	–	P	Yes	PVS1, PM2, PM4

PD, probably damaging; D, deleterious; N, neutral; P, pathogenic; LP, likely pathogenic; PolyPhen2 result, the score is closer to 1, the damaging will be more strong; Proven Result, variants with a score equal to or below − 2.5 are considered “deleterious”, variants with a score above − 2.5 are considered “neutral”

### The copy number variation verification of *STRC*

**Fig. 1:**
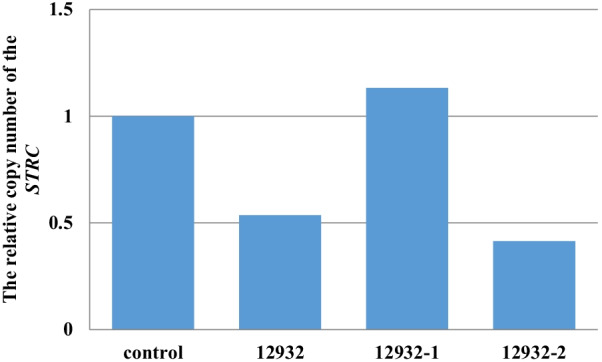
The real time PCR verification of the *STRC* copy number variant

The target NGS showed that there was a heterozygous deletion of *STRC* in proband 12932. Quantitative RT-PCR was performed to estimate *STRC* copy number in members of proband 12932’s family and in healthy people. Each sample was assayed in triplicate for each gene using SYBR Green PCR Master Mix and a StepOnePlus Real Time PCR System. The primers used to amplify *STRC* and the internal reference gene *GAPDH* were showed in Table 4. The *STRC* copy number was calculated by dividing the yield of the *STRC* gene by that of the reference gene. The amplification conditions were: 95 °C for 3 min, then 40 cycles of 95 °C for 15 s, 60 °C for 30 s, then 50 °C for 15 s. The relative quantitative analysis Cq value was determined using the 2−△△Ct method to calculate the relative *STRC* copy number in the family members and healthy people. The results showed that the relative copy number of *STRC* in proband 12932 and his mother (12932-2) was only approximately 50% of that of a normal person (Fig. 1).

**Table 4:** Primer sequences for RT-PCR

Primer’s name	Primers		Length
*STRC*	Forward	AGTAAGTCCACCTTTACCTCAG	81 bp
	Reverse	TCCAGCACATCAGCAGTT	
*GAPDH*	Forward	CGGAGTCAACGGATTTGGTCGTAT	308 bp
	Reverse	AGCCTTCTCCATGGTGGTGAAGAC

## Discussion

In this study, we performed a variant analysis of 92 unrelated Chinese NSHL patients. We determined a molecular diagnosis in 18 probands, with 33 different variants in 14 deafness genes (Table 2). We identified 18 novel variants in 12 deafness genes, which were not previously reported in ClinVar or HGMD. According to the ACMG guidelines and InterVar software, 10 variants of them were categorized as “pathogenic” variant, and 8 were categorized as “likely pathogenic” variants (Table 3).

Among the 18 probands who received a genetic diagnosis, 15 were autosomal recessive, two were autosomal dominant, and one was X/Y-linked. Yang et al. [23] recruited 190 NSHL patients, and after excluding the common *GJB2*, *SLC26A4* and *MT-RNR1* variants, 33 probands were determined to have rare HL variants, 28 were autosomal recessive, four were autosomal dominant, and one was mitochondrial. The number of autosomal recessive patients was much lower than in our study, which might be caused by regional differences. In our study, variants in *TMC1*, *CDH23*, *LOXHD1* and *USH2A* were each detected in two probands, while variants in *POU3F4*, *OTOA*, *GPR98*, *GJB6*, *TRIOBP*, *SLC26A4*, *MYO15A*, *TNC*, *STRC* and *TMPRSS3* were each detected in one proband. Among the 33 rare HL cases reported by Yang et al., the most frequently detected variant was in *MYO15A* (four times), then in *TMC1*, *USH2A*, *PCDH15*, and *GPR98* (three times each) [23]. Although the detection rates of the *TMC1* and *USH2A* variants were high in both this study and that of Yang et al., we detected an *MYO15A* variant in only one case, while Yang et al. did not detect any *LOXHD1* variants, which we detected twice in our patients. These differences may be caused by regional differences between north and south China. Of course, this may also be caused by the sample size not being large enough.

Some deafness gene screening techniques can screen for hot-spot variants in *SLC26A4*. However, targeted screening tests might miss rare variants of *SLC26A4*. In patient 12751, we detected a compound heterozygous variant, c.589G>A/ c.1238A>G, which was not in the variant hot-spots of *SLC26A4*. Therefore, for patients with deafness, it is best not to use deafness gene screening technology. Targeted sequencing technology or whole exome sequencing technology should be used for diagnosis.

CNV is one of the main forms of structural genome variation, and is a cause of many genetic diseases. NGS is increasingly used to test for CNVs in many diseases. In patient 12932, we detected a CNV (a heterozygous deletion) in *STRC,* which has been previously reported [27, 28]. *STRC* CNV is common in HL patients [31] and 72 types of deletion and 35 duplications of *STRC* are included in the ClinVar database. Targeted-NGS methods to detect CNVs in HL patients can still be improved, for example specificity and sensitivity can be enhanced; however, whole exome sequencing (WES) or whole-genome sequencing (WGS) are recommended to detect CNV in HL patients.

In our study, we identified 18 novel variants in 12 deafness genes. These variants included eight missense variants, four nonsense variants, five frameshift variants and 1 splice site variants (Table 3). The nonsense variants and frameshift variants caused the peptide chain to terminate prematurely, which shortened the length of the peptide chain, and then affected the function of the gene. We use PolyPhen2 and PROVEAN tools to assess the possible functional role of the eight novel missense variant. The missense variants c.805C>T, c.5957T>C and c.6830C>A of CDH23, c.774A>C of OTOA were assessed as probably damaging by PolyPhen2, however, these four missense variants were assessed as neutral by PROVEAN. Then we checked the conservative of these four missense variants, all the four variant were highly conserved in different species. Combined with ACMG guidelines and InterVar software, we speculated that these four missense variants were “likely pathogenic” variants.

## Conclusions

We used targeted-NGS for genetic diagnosis of 18 NSHL probands. We identified 18 novel variants in 12 deafness genes, which enlarged the variant spectrum of deafness genes in the Han Chinese population. These findings help inform the genetic diagnosis of deafness and add to the theoretical basis for the prevention of deafness. However, 74 patients in our cohort did not receive a clear genetic diagnosis; therefore, further WES or WGS testing is needed to identify mutations in other HL-causing genes or to discover new disease-causing genes for these patients.

## Data Availability

The raw sequence data reported in this paper have been deposited in the Genome Sequence Archive (Genomics, Proteomics & Bioinformatics 2021) in National Genomics Data Center (Nucleic Acids Res 2021), China National Center for Bioinformation / Beijing Institute of Genomics, Chinese Academy of Sciences (GSA-Human:HRA001546) that are publicly accessible at https://bigd.big.ac.cn/gsa-human/browse/HRA001546.

## Abbreviations

ACMG: American College of Medical Genetics and Genomics
ASSR: Auditory steady-state evoked response
CNV: Copy number variation
HGMD: Human Gene Variant Database
HL: Hearing loss
NSHL: Non-syndromic hearing loss
NGS: Next-generation sequencing
WES: Whole exome sequencing
WGS: Whole-genome sequencing
